# PTPRH promotes the progression of non-small cell lung cancer via glycolysis mediated by the PI3K/AKT/mTOR signaling pathway

**DOI:** 10.1186/s12967-023-04703-5

**Published:** 2023-11-16

**Authors:** Shu Wang, Zhiming Cheng, Yan Cui, Shuoyan Xu, Qiu Luan, Shan Jing, Bulin Du, Xuena Li, Yaming Li

**Affiliations:** Department of Nuclear Medicine, The First Hospital of China Medical University, No. 155, Nanjing Northern Street, Shenyang, 110001 Liaoning People’s Republic of China

**Keywords:** Non-small cell lung cancer, Protein tyrosine phosphatase H receptor, Glycolysis, F-18-fluorodeoxyglucose, Positron emission tomography, Computed tomography

## Abstract

**Background:**

The protein tyrosine phosphatase H receptor (PTPRH) is known to regulate the occurrence and development of pancreatic and colorectal cancer. However, its association with glycolysis in non-small cell lung cancer (NSCLC) is still unclear. In this study, we aimed to investigate the relationship between PTPRH expression and glucose metabolism and the underlying mechanism of action.

**Methods:**

The expression of PTPRH in NSCLC cells was evaluated by IHC staining, qRT‒PCR and Western blotting. The effect of PTPRH on cell biological behavior was evaluated by colony assays, EdU experiments, Transwell assays, wound healing assays and flow cytometry. Changes in F-18-fluorodeoxyglucose (^18^F‐FDG) uptake and glucose metabolite levels after altering PTPRH expression were detected via a gamma counter and lactic acid tests. The expression of glycolysis-related proteins in NSCLC cells was detected by Western blotting after altering PTPRH expression.

**Results:**

The results showed that PTPRH was highly expressed in clinical patient tissue samples and closely related to tumor diameter and clinical stage. In addition, PTPRH expression was associated with glycometabolism indexes on ^18^F-FDG positron emission tomography/computed tomography (PET/CT) imaging, the expression level of Ki67 and the expression levels of glycolysis-related proteins. PTPRH altered cell behavior, inhibited apoptosis, and promoted ^18^F-FDG uptake, lactate production, and the expression of glycolysis-related proteins. In addition, PTPRH modulated the glycometabolism of NSCLC cells via the phosphatidylinositol-3-kinase (PI3K)/protein kinase B (AKT)/mammalian target of rapamycin (mTOR) signaling pathway, as assessed using LY294002 and 740Y-P (an inhibitor and agonist of PI3K, respectively). The same results were validated in vivo using a xenograft tumor model in nude mice. Protein expression levels of PTPRH, glycolysis-related proteins, p-PI3K/PI3K and p-AKT/AKT were measured by IHC staining using a subcutaneous xenograft model in nude mice.

**Conclusions:**

In summary, we report that PTPRH promotes glycolysis, proliferation, migration, and invasion via the PI3K/AKT/mTOR signaling pathway in NSCLC and ultimately promotes tumor progression, which can be regulated by LY294002 and 740Y-P. These results suggest that PTPRH is a potential therapeutic target for NSCLC.

**Graphical Abstract:**

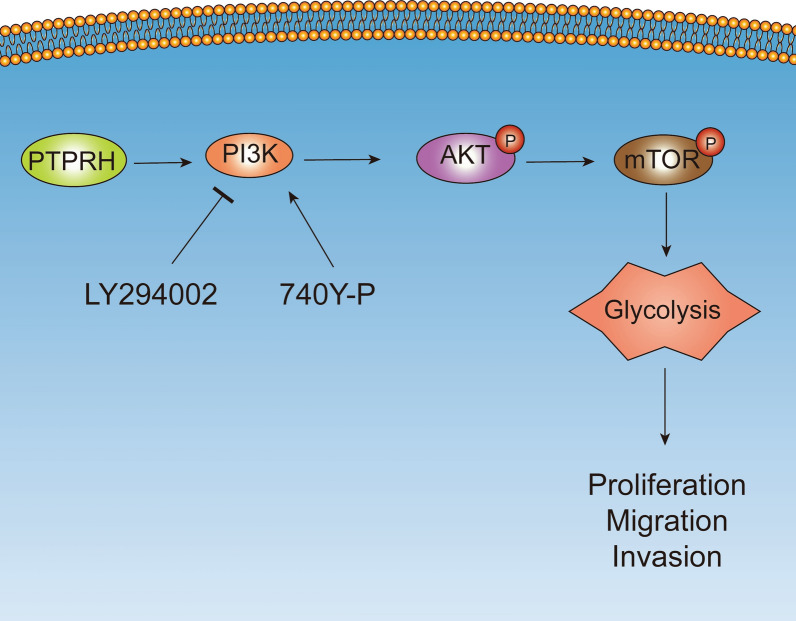

**Supplementary Information:**

The online version contains supplementary material available at 10.1186/s12967-023-04703-5.

## Background

The morbidity and mortality associated with non-small cell lung cancer (NSCLC) are increasing [1]. Despite advances in the diagnosis and treatment of patients with NSCLC, significant difficulties related to clinical treatment still exist [2]. Therefore, there is a crucial need to conduct in-depth research on the mechanisms of NSCLC lesion progression and explore new key targets; for example, targeted energy metabolism therapy for tumors has received considerable attention and has potential clinical significance [3].

Aerobic glycolysis is a crucial characteristic of energy metabolism in tumor cells. Under aerobic conditions, malignant tumors obtain adenosine triphosphate via glycolysis; this metabolic phenomenon is called aerobic glycolysis, also known as the “Warburg effect” [4]. This effect is also the molecular biological basis for the use of F-18-fluorodeoxyglucose positron emission tomography/computed tomography (^18^F‐FDG PET/CT) in oncology [5, 6]. ^18^F‐FDG PET/CT is a powerful imaging tool that provides valuable functional information based on the increased glucose uptake and glycolysis of cancer cells. The main mechanisms of ^18^F‐FDG uptake involve increased expression of glucose transporters on the cell membrane and rate‐limiting glycolytic enzymes. Previous studies have shown that GLUT1 and GLUT3 are highly expressed in various cancers and can transport glucose and ^18^F-FDG  [7]. Hexokinase is the first rate-limiting enzyme in cell glycolysis and can further phosphorylate ^18^F-FDG in cells [8]. As a key rate-limiting enzyme in the glycolytic pathway, PKM2 plays a crucial role in tumor cell metabolism and proliferation [9]. LDHA catalyzes the conversion of pyruvate to lactate and is a key enzyme in glycolysis [10]. Therefore, changes in glycolysis in NSCLC are reflected by changes in the expression of GLUT1, HK2, PKM2, and LDHA. Protein kinase B (AKT) is known as "Warburg kinase," and it promotes tumor cell metabolic reprogramming and increases cell invasiveness [11]. The phosphatidylinositol-3-kinase (PI3K)/AKT/mammalian target of rapamycin (mTOR) signaling pathway has multiple functions related to the regulation of various biological behaviors in tumor cells and plays a crucial role in the occurrence and development of NSCLC [12, 13]. Tumor cells obtain a large amount of energy via glycolysis while also providing intermediate products for other metabolic pathways. Cutting off the energy source should be the first step in blocking tumor growth. Therefore, finding key targets for the regulation of NSCLC aerobic glycolysis is a feasible strategy for treating tumors and an important means of improving prognosis. The combination of immunotherapy, chemotherapy, and aerobic glycolysis-targeted therapy is a very promising tumor treatment approach for clinical practice.

The dynamic balance between protein phosphorylation and dephosphorylation is controlled by the activity of receptor tyrosine kinases (RTKs) and protein tyrosine phosphatases (PTPs). This abnormal change in enzyme activity balance play critical roles in tumorigenesis [14, 15]. RTKs are recognized as carcinogenic factors for various types of malignant tumors [16, 17]. Carcinogenic RTKs can activate downstream signaling pathways and mediate tumor cell metabolic reprogramming via different mechanisms, thereby affecting glycolysis in tumors [15, 17–20]. In recent years, targeted therapy based on RTKs has shown promise after preliminary use in clinical practice. However, clinical recurrence is also common due to acquired resistance to these molecular therapies [21]. PTPs which play a crucial role in this collaborative process, can directly control the activation of RTKs or indirectly regulate the signaling pathway downstream of RTKs by playing positive or negative roles to tumor progression. Thus, PTPs play a crucial role in cancer development [22]. The most common mutation in the PTP family occurs in the gene that encodes the protein tyrosine phosphatase H receptor (PTPRH) [23]. PTPRH is mainly expressed in gastrointestinal epithelial cells and is localized in the microvilli of these cells, indicating that PTPRH may play a role in maintaining microvillus structure [24]. Recent studies have shown that PTPRH regulates the occurrence and development of colorectal cancer and NSCLC [24–26]. PTPRH plays an increasingly important role in the progression of different tumors by regulating cell proliferation, migration, and invasion [27]. However, the correlation of PTPRH expression with glycolysis in NSCLC remains unclear. In this study, we aimed to investigate the relationship between PTPRH expression and glucose metabolism and the underlying mechanism of action. Based on a literature search, we found that PTPRH is highly expressed in NSCLC and is closely associated with poor prognosis. In addition, gene set enrichment analysis (GSEA) demonstrated that PTPRH is highly enriched in the PI3K/AKT/mTOR pathway, suggesting that PTPRH may affect the cell cycle, apoptosis, and glycolysis through the PI3K/AKT/mTOR signaling pathway. Our findings indicate that PTPRH promotes NSCLC progression and may be an important prognostic marker of metastatic and advanced NSCLC.

## Methods

### Bioinformatics analyses

The clinical data and mRNA sequences of 351 patients with NSCLC were obtained from The Cancer Genome Atlas (TCGA) (https://cancerge-nome.nih.gov/). In addition, we analyzed the Gene Expression Omnibus (GEO) expression profiles of GSE31210 (90 NSCLC samples) and GSE30219 (300 NSCLC samples). We collected data on PTPRH expression and the integrin family for each case.

The differences in function between the gene expression sets of the two biological states were evaluated using GSEA (GSEA v2.2.3; www.broadinstitute.org/gsea). With annotations based on the Kyoto Encyclopedia of Genes and Genomes (KEGG), GSEA was conducted on NSCLC data from TCGA to investigate the metabolic pathways involved in PTPRH in cells. To evaluate statistical significance, a false discovery rate < 0.25 and* P* < 0.05 were utilized as threshold values.

### Patients and clinical information

Eighty patients (45 men and 35 women; mean age, 59.70 ± 9.49 years) with NSCLC were included in this study. All patients underwent ^18^F-FDG PET/CT before treatment at the Department of Nuclear Medicine, the First Hospital of China Medical University, between June 2018 and August 2020. The inclusion criteria were as follows: (1) ^18^F-FDG PET/CT imaging was performed between June 1, 2018, and August 30, 2020; (2) lung cancer resection surgery was performed within 1 month after imaging, NSCLC was diagnosed post-surgery, and the pathological specimen obtained during the surgery contained adjacent lung tissue; and (3) on the day of imaging, a fingertip blood sugar measurement after 6 h of fasting and before ^18^F-FDG injection was below 11 mmol/L. Patients were excluded based on the following criteria: (1) they were diagnosed with other malignant tumors; (2) they had undergone immunotherapy, chemotherapy, or radiation therapy before ^18^F-FDG PET/CT imaging and surgery; or (3) they had accompanying pulmonary diseases such as tuberculosis or inflammation. All procedures involving human studies were approved by the Ethics Committee of the First Hospital of China Medical University (IRB number: AF-SOP-07-1.1-01). Demographic and clinical characteristics were recorded and analyzed. All the participants signed a written informed consent form.

### ^18^F-FDG PET/CT imaging and data analysis

After 6 h of fasting, the patients were administered an i.v. injection of ^18^F‐FDG (3.7 MBq/kg), and after 60 min, PET/CT images were acquired using an integrated PET/CT scanner (Biograph mCT; Siemens, Germany). True D software was used to open PET/CT images on the Syngo multimedia workplace workstation using a 50% threshold value. This software automatically outlines the region of interest and extracts indicators, including the maximum and mean standard uptake values, i.e., SUVmax and SUVmean, respectively, which are calculated automatically, followed by a metabolic tumor volume (MTV) measurement. Two nuclear medicine experts extracted the relevant parameters based on the image analysis method, i.e., total lesion glycolysis (TLG) = SUVmean × MTV. Each index was measured three times to obtain an average value. Each indicator was measured three times, followed by calculation of the average value.

### Antibodies and reagents

A rabbit anti-PTPRH (NBP2-20005) antibody was purchased from Novus Biologicals (Colorado, USA). Rabbit anti-GLUT1 (ab115730) and rabbit anti-HK2 (ab209847) were purchased from Abcam (Cambridge, MA, USA). Rabbit anti-PKM2 (#4053), rabbit anti-LDHA (#3582), rabbit anti-Akt (#9272), rabbit anti-phospho-Akt (Ser473) (#9271), rabbit anti-phospho-PI3K (Thr202/Tyr204) (#4370), rabbit anti-PI3K (#4257), rabbit anti-mTOR (#2983), rabbit anti-phospho-mTOR (#5536), and mouse anti-β-actin (#3700) were purchased from Cell Signaling Technology (Danvers, MA, USA). Matrigel was purchased from Corning Inc. (Corning, NY, USA). The primary antibody concentration used for Western blotting was 1:1000. The secondary antibody concentration used for Western blotting was 1:2000. The antibody concentration used for IHC was 1:200.

### Immunohistochemical analyses

Eighty NSCLC lesions and 40 adjacent lung tissue sections were immunostained with an anti-PTPRH antibody, and 80 NSCLC sections were immunostained for glycolysis-related proteins, i.e., glucose transporter type 1 (GLUT1), hexokinase 2 (HK2), pyruvate kinase M2 (PKM2), lactate dehydrogenase A (LDHA) and Ki67, using the EliVision two-step staining method. Xenograft tumors were immunostained for the PTPRH, Ki67, GLUT1, LDHA, PI3K, phospho-PI3K (p-PI3K), AKT, and phospho-AKT (p-AKT) protein. Staining was evaluated by scanning the entire tissue specimen under high magnification (×20). Protein expression was visualized and classified based on the percentage of positive cells and the staining intensity. Five fields of view were selected randomly from each section.

The experimental methods used for immunohistochemical staining have been described previously [28]. The scoring criteria for immunohistochemical staining were as follows: the staining intensity score was 0–3 points, with colorless, light yellow, brown, and dark brown corresponding to 0, 1, 2, and 3 points, respectively. The percentage of positive cells in the same field of view was divided into 1–4 points. Cell percentages of < 5, 5–25, 25–50, 50–75, and > 75% corresponded to 0, 1, 2, 3, and 4 points, respectively. The two scores were multiplied to obtain the visual field score, and the final score of the pathological section was the average of the scores of the five fields of view.

### Cell lines

The H1299, HCC827, A549, Calu-1, H460, H292, and PC9 human NSCLC cell lines and the human bronchial epithelial (HBE) cell line were obtained from the Chinese Academy of Sciences (Beijing, China). A549 cells were grown in Dulbecco’s modified Eagle’s medium (DMEM; HyClone, Logan, UT, USA), whereas H1299, HCC827, Calu-1, H460, H292, PC9, and HBE cells were grown in Roswell Park Memorial Institute 1640 medium (HyClone) supplemented with 10% fetal bovine serum (FBS; BI, Kibbutz Beit-Haemek, Israel) at 37 °C and 5% CO_2_.

### RNA interference

PTPRH-specific siRNAs (si1 and si2) and negative control (NC) siRNA were purchased from ViewSolid Biotech (Beijing, China). The coding strand sequence of human PTPRH si1 was 5′-GCUCAAGAAGUCACUGAAATT-3′ and that of PTPRH si2 was 5′-GCCUCUGAUGAAUGAUGAATT-3′. Cells were transfected with siRNAs using Lipofectamine 2000 (Invitrogen, Carlsbad, CA, USA) following the manufacturer’s protocol.

### Lentiviral construction and transfection

Design and construction of a lentivirus for PTPRH overexpression with green fluorescent protein (GFP) expression were performed by Obio Technology Corp., Ltd. (Shanghai, China). A549 and NCI-H460 cells were transfected with lentiviral vectors following the manufacturer’s instructions. The control cells were transfected with an empty vector expressing GFP. PTPRH-overexpressing cells were named OE cells, and empty vector cells, i.e., NC cells, were used as controls.

### RNA isolation and real-time polymerase chain reaction (RT‒PCR)

TRIzol reagent (Invitrogen) was used to isolate total RNA from the cultured cells. The concentration of total RNA was quantified by measuring the absorbance at 260 nm. The PrimeScript™ RT Reagent Kit (Takara, Japan) was used for mRNA reverse transcription. Real-time polymerase chain reaction was performed using SYBR Premix Ex Taq II (TaKaRa) and an Applied Biosystems® 7500 RT‒PCR System (Thermo Fisher Scientific, Waltham, MA, USA) with 18S rRNA as an internal control. The 2 − ΔΔCt method was used to calculate the fold change in the RNA expression of one sample compared to the calibration sample. The primer sequences are listed in Table 1.

**Table 1:** Sequences of primers for qRT-PCR

Gene	Forward (5′–3′)	Reverse (5′–3′)
PTPRH	GCCATAGCCGAGTGTGAGAC	CAACTCCACAAGAGGTACATCG
18S	CCCGGGGAGGTAGTGACGAAAAAT	CGCCCGCCCGCTCCCCAAGAT

### Western blotting

The cells were lysed in a buffer containing 1% Triton X-100. Protein lysates were separated by SDS‒PAGE and transferred to a polyvinylidene fluoride (PVDF) membrane. The PVDF membranes were blocked using 5% skim milk, followed by incubation with the indicated primary antibodies overnight at 4 °C. After the appropriate secondary antibodies were added, proteins were detected using an enhanced chemiluminescence reagent and visualized using an electrophoresis Gel Imaging Analysis System (DNR Bio-Imaging Systems, Jerusalem, Israel).

### Colony formation assay

Cell viability was determined using the colony formation assay. After transfection, cells were seeded at a density of 500 cells/well in 6-well plates and cultured for 14 days at 37 °C in a 5% CO_2_ atmosphere. After 14 days, the cells were fixed in 4% paraformaldehyde and then stained with crystal violet.

### 3-(4,5-dimethylthiazol-2-yl)-2,5-diphenyltetrazolium bromide (MTT) assay

Selected cells were incubated in 96-well plates for 24 h, and then 20 µL of the MTT reagent (5 mg/L) was added per well. After incubation for another 4 h, the cells were centrifuged. The supernatant was removed, and 200 µL of dimethylsulfoxide was added to the pelleted cells. The absorbance was measured at 570 nm.

### 5-Ethynyl-2′-deoxyuridine (EdU) assay

Selected cells were incubated in 96-well plates for 24 h. Then, 50 μM EdU (RiboBio, Guangzhou, China) was added to each well, and the cells were incubated at 37 °C for 2 h before being fixed with formaldehyde for 30 min and incubated with glycine for 5 min. After being washed with PBS, the cells were reacted with 100 μL of 1 × Apollo reaction cocktail for 30 min, followed by incubation with 1 × Hoechst 33342 (5 g/mL) to stain nuclei.

### Scratch assay

Selected cells were seeded in 12-well plates. After the cells were fully grown, the culture medium was discarded and replaced with PBS. The cells were washed 3 times with PBS after scratching with a 200 µL pipette tip, incubated with 2% FBS for a predetermined time and then observed under a microscope (Olympus, Tokyo, Japan) and analyzed statistically.

### Transwell assay

The Transwell assay was performed in 8 µm Transwell chambers. The filters were precoated with Matrigel to form a genuine reconstituted basement membrane, followed by the addition of culture medium containing 10% FBS to the lower chambers. Next, 2 × 10^4^ H460 cells/200 µL and 5 × 10^4^ A549 cells/200 µL were placed in the upper chambers. After 24 h, cells remaining on the upper membrane were removed with a cotton swab, whereas cells that had invaded through the membrane were fixed in methanol and stained with Wright–Giemsa. The cells were then observed under a microscope (Olympus) and statistically analyzed.

### Flow cytometry assay

The selected cells were treated for 12 or 24 h, and then, the cells were double-stained with annexin V-fluorescein isothiocyanate (2 μL) and propidium iodide (PI) (4 μL) for 15 min each in the dark for the apoptosis experiment. The cells were harvested and fixed with 70% (v/v) cold ethanol at 4 °C overnight for the cell cycle test. After 30 min of incubation with 100 µg/mL RNase A and 10 μg/mL PI staining solution in the dark, the cells were analyzed using a FACScan flow cytometer (Becton Dickinson, USA). The data were analyzed using Cell Quest (USA) and FlowJo software.

### Xenograft studies and micro-PET scans

Four-week-old female BALB/c nude mice (20–25 g) were purchased from Beijing Vital River Laboratory Animal Technology Co. Ltd. (Beijing, China). The animals were housed in a specific pathogen-free environment at the Animal Laboratory Unit of China Medical University. All in vivo experiments were performed following the guidelines of the Institutional Review Board of China Medical University (IACUC Issue No. 2023017). Following the recommendations of the Institutional Animal Care and Use Committee (IACUC) of China Medical University, the mice were euthanized by CO_2_ inhalation when the tumor diameter reached 15 mm. The untransfected A549 cells (3 × 10^6^) were suspended in 150 μL PBS and then injected subcutaneously into the right scapular region of mice. However, A549 cells (3 × 10^6^) expressing empty vector carrying short hairpin RNA-NC (sh-NC) or PTPRH knockdown lentivirus (sh-PTPRH) were injected subcutaneously into the dorsal flanks of mice. Measurements were recorded once every 3 days after tumor formation. The metabolic level of each tumor was measured using micro-PET scans (Madic, Shandong, China) with ^18^F-FDG. After 6 h of fasting, the mice were injected with a radiolabeled probe (370 kBq/g) via the lateral tail vein. After 30 min, static PET images were collected under 2% isoflurane anesthesia. All procedures were supervised and inspected by the IACUC and the laboratory animal department.

### Assay of ^18^F-FDG uptake

Cell uptake experiments were performed using ^18^F‐FDG to assess glucose uptake to examine changes in cell metabolism. The selected cells were cultured in 6‐well cell plates. After 48 h, the cells were washed 3 times with phosphate-buffered saline (PBS) and incubated in 2 mL of DMEM. Then, ^18^F‐FDG (148 kBq [4 Ci/mL]) was added to each well, and the cells were incubated for 60 min at 37 °C. Whole‐cell lysates were produced using 1 mL of trypsin‐ethylenediaminetetraacetic acid, and radioactivity was measured using a γ‐counter (ZonKia, Hefei, China).

### Cell metabolism assay

Lactate levels were measured in the cell culture medium to examine changes in cellular glucose metabolites. The selected cells were cultured in 6‐well cell plates. After 48 h, the cells were washed, centrifuged, and lysed, and lactate concentrations were measured using a Lactate Assay Kit (KeyGEN, Nanjing, China) following the manufacturer’s instructions. The optical density was measured at 530 nm using a microplate reader (Thermo Fisher Scientific, Waltham, MA, USA).

### Statistical analyses

Measurements were recorded from three independent experiments, and the data are shown as the mean ± S.D. Correlation analysis was conducted using *Spearman* correlation analysis, and two-sample *t* tests were used for intergroup comparisons. *P* < 0.05 indicates a statistically significant difference. All statistical analyses were conducted using SPSS statistical software package (version 26.0), GraphPad Prism 8, and R software (version 3.3.3).

## Results

### High expression of PTPRH predicts poor prognosis for NSCLC patients

**Fig. 1:**
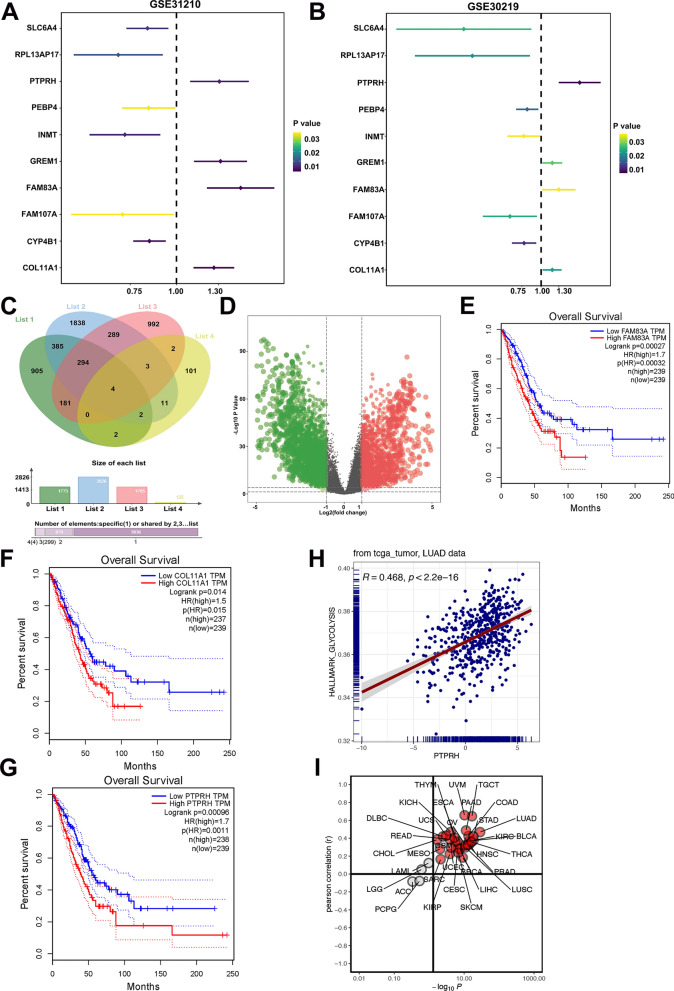
*PTPRH* is highly expressed in NSCLC and is closely related to poor prognosis. **A**, **B** One-way Cox analysis of 10 genes with prognostic value in two GEO-independent datasets (GSE31210 and GSE30219). **C** Genes with prognostic value from one-way Cox analysis of TCGA-LUAD data were identified by intersection with genes in the GEO dataset. **D** Analysis of differentially expressed genes in TCGA-LUAD, presented as volcano plots. **E**–**G** Correlations of *FAM83A*, *COL11A1*, and *PTPRH* expression with survival prognosis. **H** Correlation analysis of *PTPRH* expression with glycolysis. **I** Correlation analysis of *PTPRH* expression with glycolysis in TCGA pancancer data. PTPRH: protein tyrosine phosphatase H receptor gene; NSCLC: non-small cell lung cancer; GEO: Gene Expression Omnibus; TCGA: The Cancer Genome Atlas; LUAD: lung adenocarcinoma

Two independent datasets were downloaded from the Gene Expression Omnibus (GEO) database, and the prognostic impact of the expression of different molecules in the tumor samples was sorted from lowest to the highest significance according to the *P* value, followed by selection of the top 10 molecules for analysis. Genes such as *FAM93A*, *COL11A1*, and *PTPRH* were significantly associated with prognosis (Fig. 1A, B), 4 genes had stable prognostic value (Fig. 1C). In addition, the expression of *FAM93A*, *COL11A1*, and *PTPRH* was significantly higher in the TCGA dataset than in normal tissues (Fig. 1D) and was significantly associated with shorter overall survival time in patients (Fig. 1E–G). Further analysis revealed that the pancancer correlation analysis of *PTPRH* was most relevant to NSCLC (Fig. 1H, I; *R*^*2*^ = 0.468, *P* < 2.2e−16).

### Correlation between PTPRH expression and ^18^F-FDG semiquantitative indicators, proliferation markers, and glycolysis-related protein expression

**Fig. 2:**
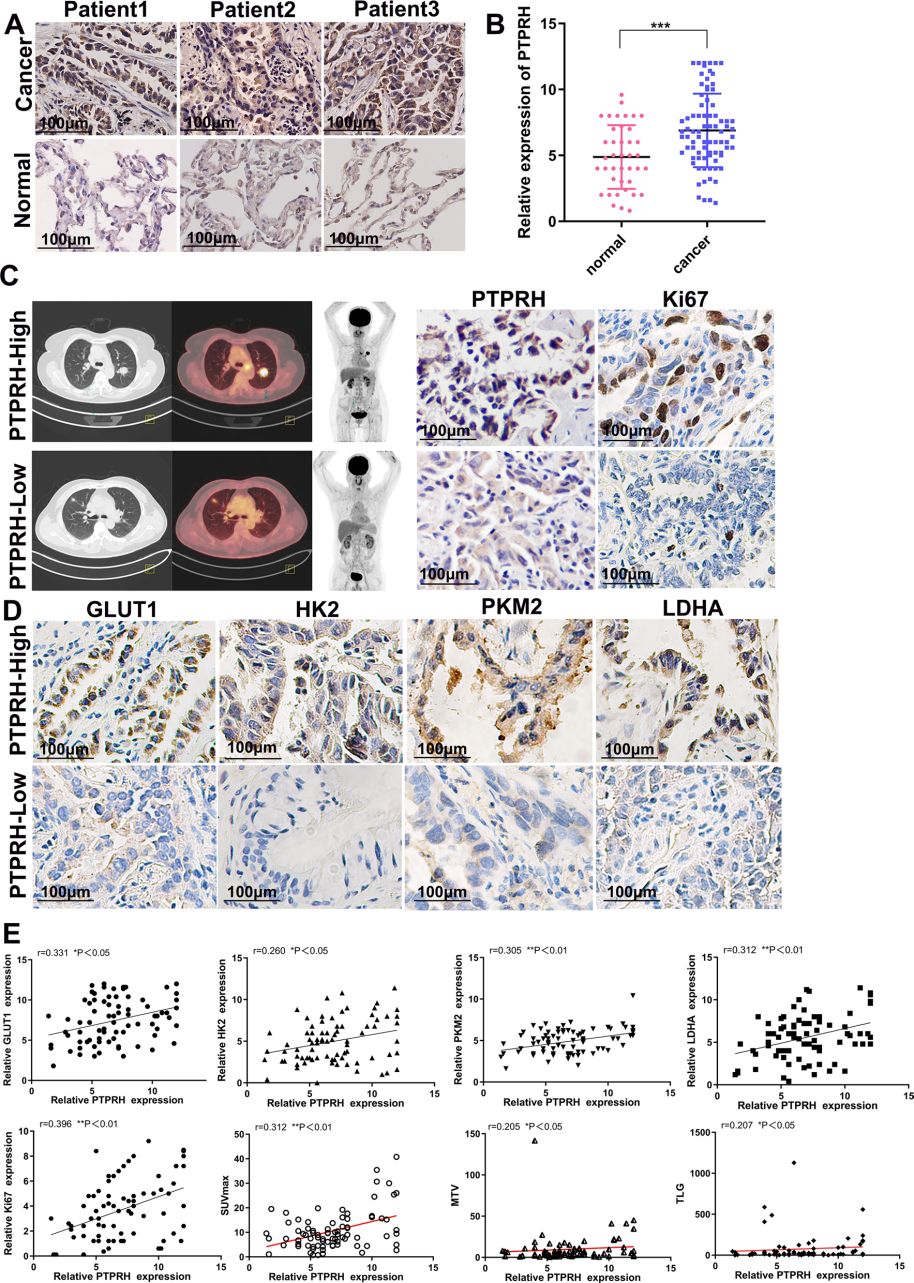
Correlation of PTPRH expression with ^18^F-fluorodeoxyglucose (^18^F-FDG) semiquantitative indicators, the expression of proliferation markers and the expression of glycolysis-related proteins. **A**, **B** Detection of PTPRH expression by immunohistochemistry (×400). **C** Correlation of PTPRH expression with ^18^F-FDG accumulation and the expression levels of Ki67. **D** In patients with high levels of PTPRH expression, immunohistochemical staining revealed high expression levels of GLUT1, HK2, PKM2, and LDHA (×400), whereas patients with low levels of PTPRH expression expressed low levels of GLUT1, HK2, PKM2 and LDHA (× 400). **E** The expression levels of PTPRH correlated positively with SUVmax, MTV, and TLG values and the expression of Ki67, GLUT1, HK2, PKM2, LDHA. ^18^F-FDG: ^18^F-fluorodeoxyglucose; GLUT1: glucose transporter type 1; HK2: hexokinase 2; PKM2: pyruvate kinase M2; LDHA: lactate dehydrogenase A; SUVmax: maximum standard uptake value

We collected 80 pathological specimens from patients with NSCLC, and immunohistochemical staining results showed that PTPRH expression was significantly higher in patients with NSCLC than in adjacent tissues (Fig. 2A, B). We retrospectively collected clinical data from 80 patients with NSCLC, grouped according to age, sex, tumor diameter, and clinical tumor stage, to compare the relationship between PTPRH expression levels and clinical characteristics. Statistical analysis showed a correlation between PTPRH expression, NSCLC tumor diameter, and clinical staging of NSCLC patients (** *P* < 0.01), but there was no significant correlation with patient age or sex (*P* > 0.05) (Table 2). In addition, our combined analysis of pathological specimens and ^18^F-FDG PET/CT results showed that the SUVmax of ^18^F-PET/CT was elevated. The tumors were relatively large in patients showing high levels of PTPRH expression by immunohistochemical staining compared with those showing low or normal levels of PTPRH expression. Similarly, Ki67 expression was higher in patients showing high levels of PTPRH expression by immunohistochemical staining than in those showing low or normal levels of PTPRH expression. Representative cases with high or low PTPRH expression and the corresponding ^18^F-FDG PET/CT images are shown (Fig. 2C). Further analysis of glycolysis-related proteins by immunohistochemical staining showed that the expression of GLUT1, HK2, PKM2, and LDHA was higher in patients with high PTPRH expression than in those with low PTPRH expression (Fig. 2D). Therefore, we further correlated the expression of PTPRH with the semiquantitative indicators of ^18^F-FDG PET/CT, immunohistochemical expression scores of proliferation markers and immunohistochemical expression scores of glycolysis-related proteins. The results showed significant positive correlations between the expression of PTPRH and the levels of GLUT1, HK2, PKM2, LDHA, and the proliferation marker Ki-67. In addition, PTPRH expression showed significant positive correlations with SUVmax, MTV, and TLG (Fig. 2E).

**Table 2:** The relationship between PTPRH expression and clinical characteristics of NSCLC patients

Clinical characteristics	N	PTPRH expression level (mean ± standard deviation)	*P* value
Age (year)			0.320
≥ 60	41	6.64 ± 2.867	
< 60	39	7.27 ± 2.737	
Gender			0.670
male	45	6.83 ± 2.828	
female	35	7.20 ± 2.807	
Tumor diameter (cm)			0.004**
≤ 3	54	6.21 ± 2.274	
> 3	26	7.94 ± 2.7363	
Clinical stage			0.003**
I–II	43	5.28 ± 2.1310	
III–IV	37	6.97 ± 2.8042	

### Downregulation of PTPRH decreased proliferation, migration, and invasion in NSCLC

**Fig. 3:**
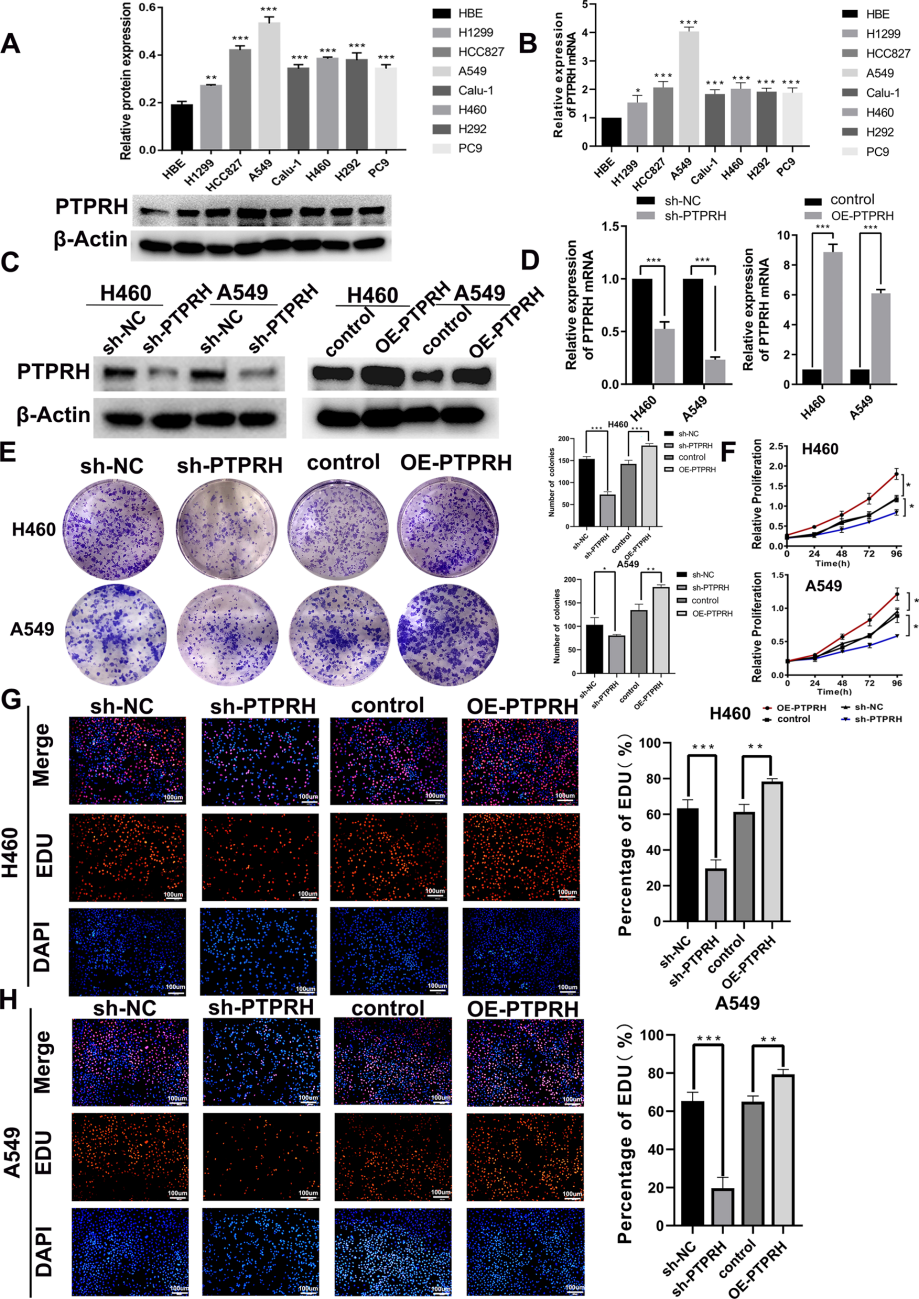
PTPRH promoted proliferation in NSCLC. **A**, **B** Detection of PTPRH expression using RT‒PCR and western blotting. **C**, **D** Expression of PTPRH after transfection by western blotting analysis and RT‒PCR. **E** The results of the colony formation assay.) **F** 3-(4,5-dimethylthiazol-2-yl)-2,5-diphenyltetrazolium bromide (MTT) assay. **G**, **H** 5-ethynyl-2′-deoxyuridine (EdU) assay show that PTPRH enhances the proliferation ability of NSCLC cells

To further explore the molecular functions of PTPRH in NSCLC, we examined the protein expression levels of PTPRH in lung cancer cell lines and found that PTPRH expression was significantly higher in tumor cells than in normal lung bronchial epithelial cells (Fig. 3A). Similar results were obtained at the mRNA level (Fig. 3B). We selected the lung adenocarcinoma cell line A549 and the large cell lung cancer cell line H460 for further analysis. We first constructed stable sh-NC, sh-PTPRH, control, and PTPRH-overexpressing (OE-PTPRH) transgenic A549 and H460 cell lines. PTPRH expression was examined after transfection with the transgenic constructs by western blotting and RT‒PCR assays (Fig. 3C, D). We examined the effect of PTPRH on the proliferation of A549 and H460 cells cultured for 14 days using a colony formation assay, which showed a significant decrease in colony formation after PTPRH knockdown and a significant increase in colony formation after overexpression of PTPRH (Fig. 3E). In addition, cell proliferation was significantly slower after knockdown of PTPRH and significantly faster after overexpression of PTPRH in both A549 and H460 cells (Fig. 3F). In addition, the results of the EdU assay further showed a significant decrease in the number of A549 and H460 cells in the DNA replication phase after PTPRH knockdown and a relative increase in the DNA replication phase after overexpression of PTPRH in both A549 and H460 cells (Fig. 3G, H).

**Fig. 4:**
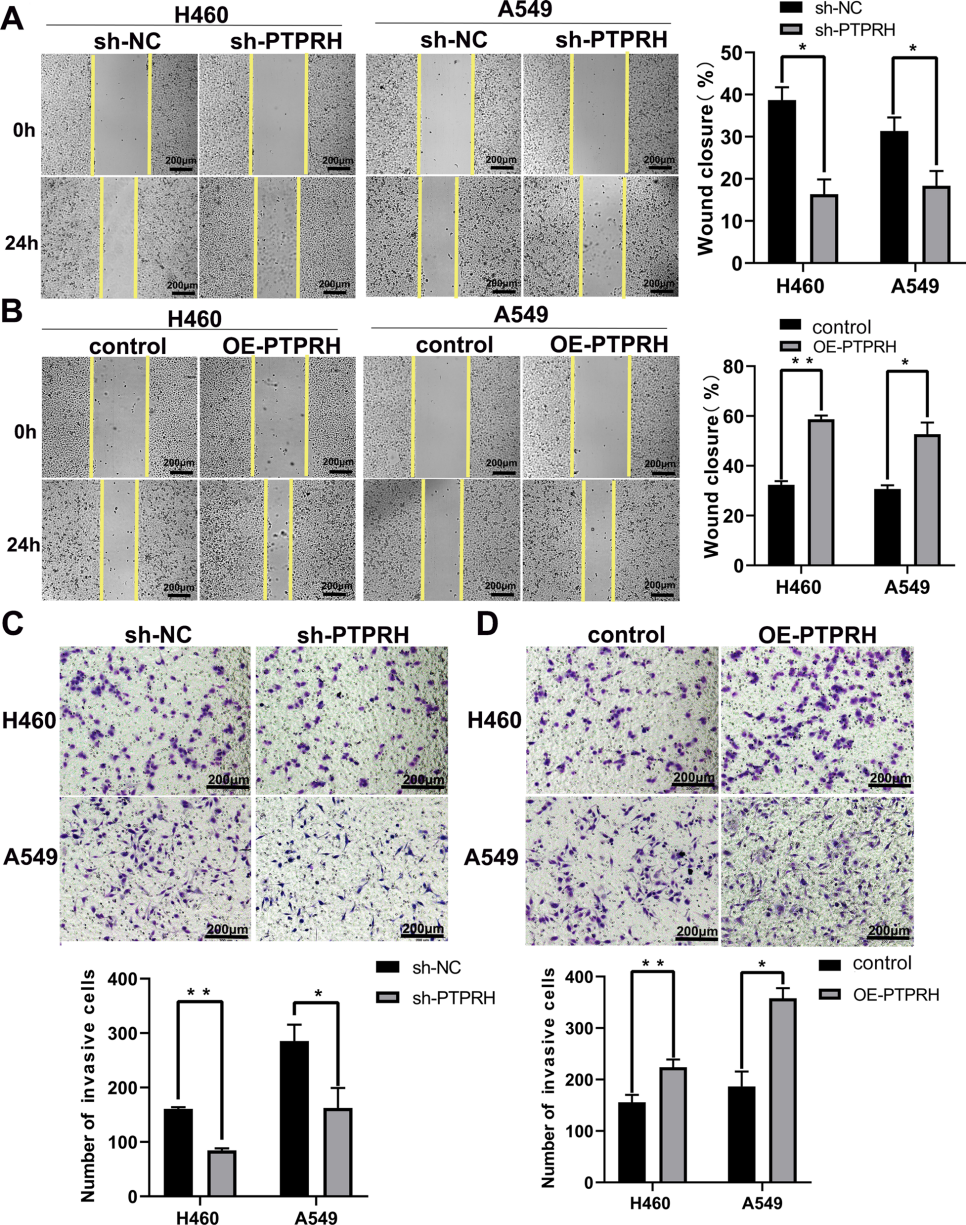
PTPRH promoted migration and invasion in NSCLC. **A**, **B** The results of the scratch assay show that PTPRH promotes the migration ability of NSCLC cells. **C**, **D** The results of the Transwell assays show that PTPRH enhances the invasion ability of NSCLC cells

Our scratch assay also demonstrated the effect of PTPRH on the migration of A549 and H460 cells, with a significant increase in the scratch area after PTPRH knockdown and a significant decrease in the cell scratch area after overexpression of PTPRH in both A549 and H460 cells (Fig. 4A, B). In addition, the results of the Transwell assay showed that the effect of PTPRH on cell invasion ability was reduced significantly after knockdown of PTPRH and enhanced after overexpression of PTPRH in both A549 and H460 cells (Fig. 4C, D).

### Downregulation of PTPRH decreased the proliferation of NSCLC by inhibiting cell cycle arrest and apoptosis

**Fig. 5:**
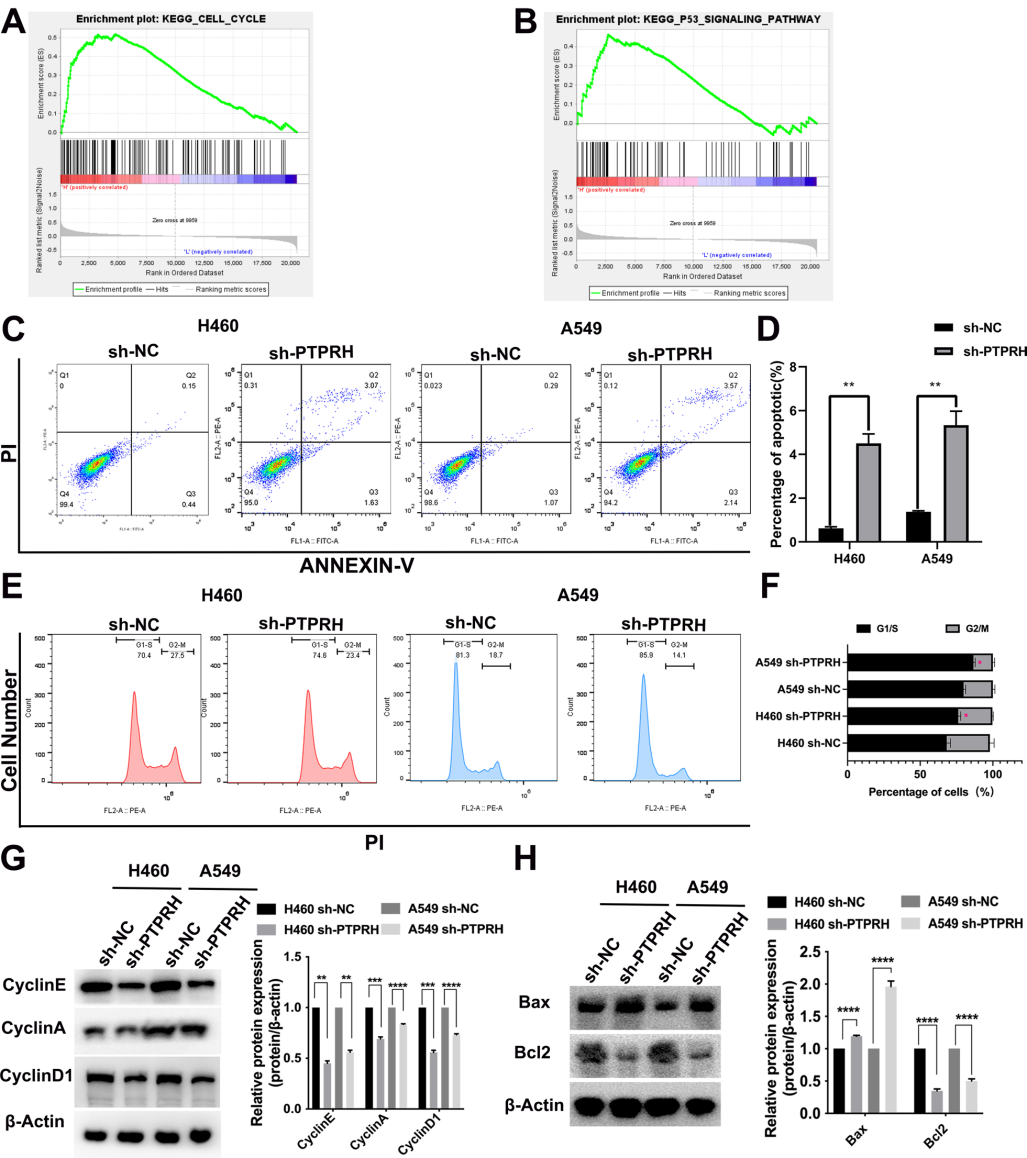
PTPRH inhibited cell cycle arrest and apoptosis. **A**, **B** Gene Set Enrichment Analysis (GSEA) of PTPRH. **C**, **D** Flow cytometry analysis of the apoptotic effect of PTPRH on NSCLC and statistical analysis. **E**, **F** Flow cytometry analysis of the effects of PTPRH expression on the cell cycle in NSCLC and statistical analysis. **G** Western blotting results showing the effects of short hairpin RNA-PTPRH (sh-PTPRH) treatment on cell cycle-related proteins. **H** Western blotting results showing the effects of sh-PTPRH treatment on the expression of apoptotic proteins

We performed a gene set enrichment analysis (GSEA) of the single-gene Kyoto Encyclopedia of Genes and Genomes (KEGG) pathway for PTPRH and found that the primary pathway positively associated with PTPRH was the cell cycle pathway; this also includes the p53 pathway, which plays a crucial role in the cell cycle. However, the strength of the association for the p53 pathway was lower than that for the cell cycle pathway (Fig. 5A, B). Flow cytometry analysis showed that more cells underwent apoptosis after PTPRH was knocked down in both A549 and H460 cells (Fig. 5C, D), and cell viability was decreased after PTPRH was knocked down in both A549 and H460 cells (Fig. 5E, F). We further examined apoptosis-related and cell cycle-related proteins and found that cyclins E, A, and D1, which are associated with the G1/S phase, were relatively downregulated after PTPRH was knocked down in both A549 and H460 cells (Fig. 5G). In addition, the apoptosis-related protein Bax was upregulated, and Bcl2 was downregulated (Fig. 5H) after PTPRH was knocked down in both A549 and H460 cells.

### PTPRH enhanced glycolysis in vivo and in vitro

**Fig. 6:**
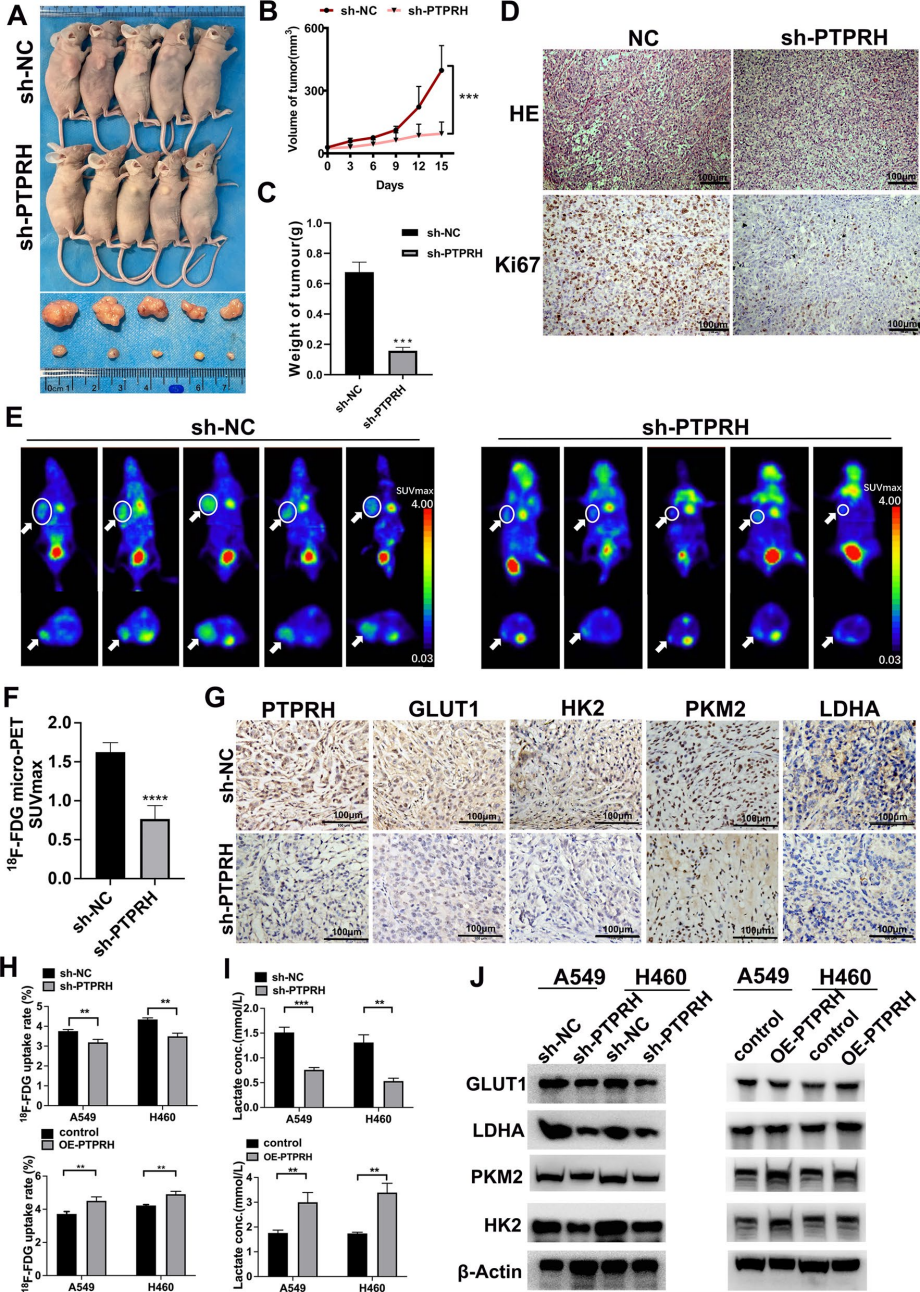
PTPRH enhanced glycolysis in vivo and in vitro. **A** Xenograft tumor sizes in the sh-PTPRH and sh-negative control (sh-NC) groups. **B**, **C** The volume and weight of tumors in the sh-PTPRH group were significantly decreased compared to those in the sh-NC group. **D** Representative hematoxylin–eosin (H&E) images of Ki67 and PTPRH staining in A549 xenografts. **E**, **F** Representative micro-PET images of mice in the sh-PTPRH and sh-NC groups. The closer the color of the tumor within the circle of mice is to red means the higher accumulation of ^18^F-FDG. The accumulation of ^18^F-FDG in the tumors of sh-NC-treated mice was significantly greater than that in the tumors of sh-PTPRH-treated mice. **G** Representative immunostained images of PTPRH, GLUT1, HK2, PKM2, and LDHA staining in A549 xenografts. **H** Cellular ^18^F‐FDG uptake was significantly decreased in the sh-PTPRH group and increased in the PTPRH-overexpressing (OE-PTPRH) group. **I** Lactate levels in the culture medium of the sh-PTPRH-treated group were significantly decreased, whereas they were increased in the OE-PTPRH-treated group. **J** Relative expression levels of glycolysis-related proteins in the sh-PTPRH and sh-NC groups by western blotting

For in vivo experiments, we used a xenograft model. Due to the fact that A549 cell line is a relatively common NSCLC cell line and exhibits a good trend in knockdown regulation of PTPRH, the A549 cell line was selected for model establishment. The tumors in the sh-PTPRH group were significantly smaller than those in the sh-NC group (Fig. 6A, B). In addition, the weight of the xenografts in the sh-PTPRH group was significantly lower than that of the xenografts in the sh-NC group (Fig. 6C). Hematoxylin–eosin staining of subcutaneous tumor tissues and immunohistochemical staining of Ki67 in mice showed that the expression of Ki67 was relatively lower than that in the sh-PTPRH group (Fig. 6D). Moreover, we examined the SUVmax of the xenograft model using micro-PET, and the results showed that the accumulation of ^18^F-FDG in the tumors of sh-NC-treated mice was significantly higher than that in tumors of sh-PTPRH mice, with a corresponding increase in SUVmax (Fig. 6E, F). In addition, immunohistochemical staining showed that PTPRH and glycolysis-related proteins (GLUT1, HK2, PKM2, and LDHA) were downregulated after PTPRH was knocked down (Fig. 6G). In the in vitro experiments, we found that the ^18^F-FDG uptake rate was lower in sh-PTPRH cells than that in sh-NC cells. Conversely, after overexpression of PTPRH, the ^18^F-FDG uptake rate was increased (Fig. 6H). Similarly, lactate levels were significantly lower after PTPRH was knocked down than that in sh-NC cells but increased significantly after overexpression of PTPRH (Fig. 6I). In addition, we verified the protein levels of glycolysis-related proteins (GLUT1, HK2, PKM2, and LDHA) by western blotting, and their expression levels were relatively decreased after PTPRH was knocked down but relatively increased after overexpression of PTPRH (Fig. 6J).

### PTPRH enhanced glycolysis via the PI3K/AKT/mTOR signaling pathway

**Fig. 7:**
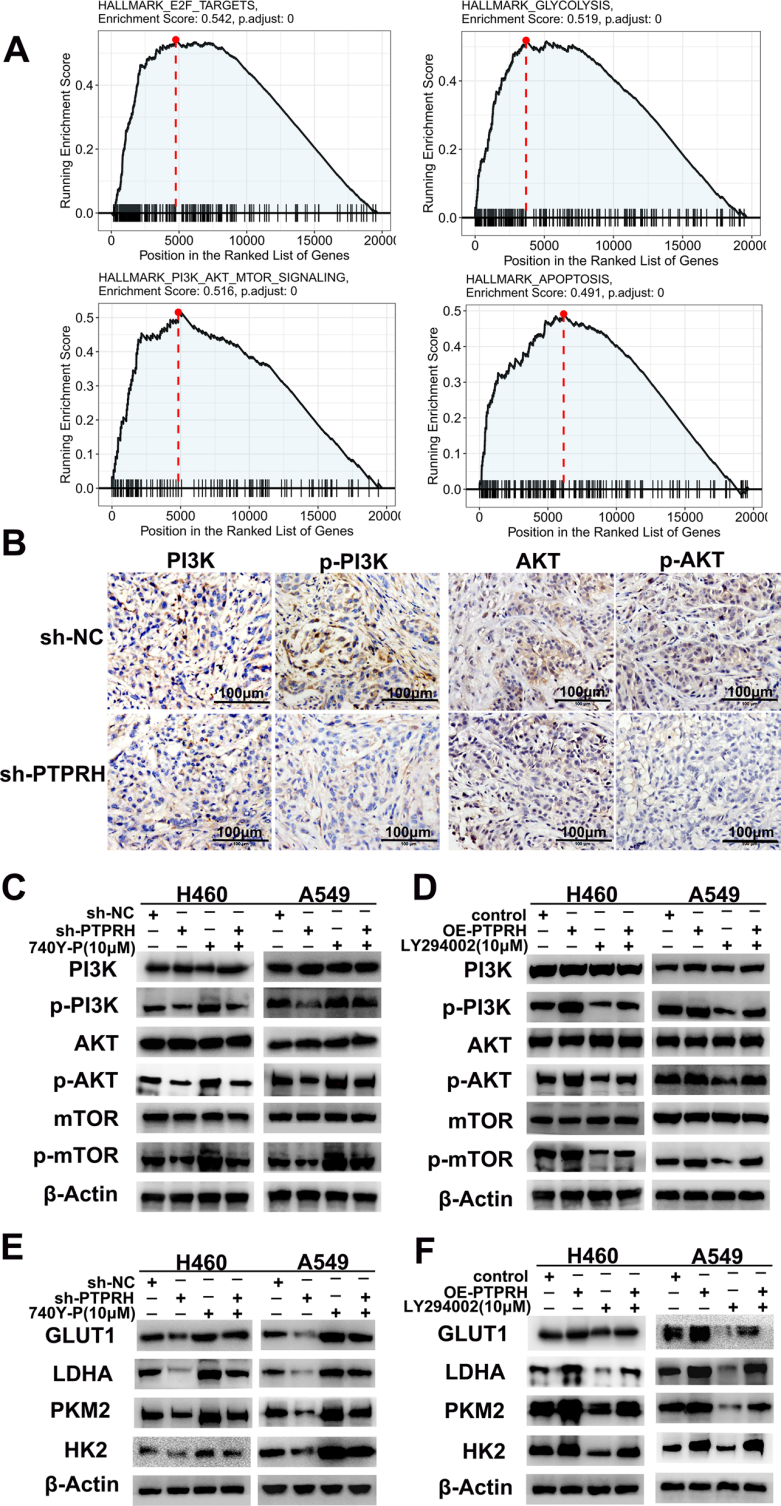
PTPRH enhanced glycolysis via the phosphatidylinositol-3-kinase (PI3K)/Protein kinase B (AKT)/mammalian target of rapamycin (mTOR) signaling pathway. **A** GSEA to identify enriched signaling pathways for PTPRH. **B** Representative immunostaining images after staining of PI3K/AKT/mTOR signaling pathway-related proteins in A549 xenografts. **C**, **D** Relative expression levels of PI3K/AKT/mTOR signaling pathway-related proteins in the sh-PTPRH and sh-NC groups by western blotting. **E** Western blotting results showing the effects of sh-PTPRH and the PI3K activator 740Y-P alone and in combination on the expression levels of glycolysis-related proteins. **F** Western blotting results showing the effects of OE-PTPRH and the PI3K inhibitor LY294002 alone and in combination on the expression levels of glycolysis-related proteins

To further explore the molecular mechanism by which PTPRH regulates glycolysis, we first performed single-gene GSEA using PTPRH (Additional file 1). The results showed that PTPRH was highly enriched in E2F (ES = 0.542; *P* = 0), glycolysis (ES = 0.519; *P* = 0), PI3K/AKT/mTOR (ES = 0.516; *P* = 0), and apoptosis (ES = 0.491; *P* = 0) in the TCGA-NSCLC database (Fig. 7A). Based on the results of GSEA, while The PI3K/AKT/mTOR signaling pathway has multiple functions in the regulation of various biological behaviors in tumor cells and plays a crucial role in the occurrence and development of NSCLC. Further validation and mechanistic studies were required to confirm the relationship between PTPRH and the PI3K/AKT/mTOR signaling pathway. We first performed immunohistochemistry experiments on tumor tissues from mice, and the results showed that p-AKT and p-PI3K were relatively downregulated in the sh-PTPRH group compared to the sh-NC group (Fig. 7B).

We performed western blotting using an inhibitor (LY294002) and an activator (740Y-P) of PI3K and found that p-PI3K, p-AKT, and phospho-mTOR (p-mTOR) were downregulated after PTPRH was knocked down in H460 and A549 cells. The downregulation of p-PI3K, p-AKT, and p-mTOR by sh-PTPRH was reversed by 740Y-P (Fig. 7C). In contrast, p-PI3K, p-AKT, and p-mTOR were upregulated after overexpression of PTPRH in both H460 and A549 cells. The upregulation of p-PI3K, p-AKT, and p-mTOR after overexpression of PTPRH was reversed by LY294002 (Fig. 7D).

We further examined the expression of glycolysis-related proteins by western blotting, which showed that GLUT1, HK2, PKM2, and LDHA were relatively downregulated after PTPRH was knocked down in H460 and A549 cells. The downregulation of GLUT1, HK2, PKM2, and LDHA expression after PTPRH knockdown was reversed by 740Y-P (Fig. 7E). In contrast, GLUT1, HK2, PKM2, and LDHA expression was relatively upregulated in both H460 and A549 cells after overexpression of PTPRH. Concomitant treatment with LY294002 and OE-PTPRH reversed the upregulated expression of GLUT1, HK2, PKM2, and LDHA observed after overexpression of PTPRH (Fig. 7F).

### Downregulation of PTPRH decreased proliferation and invasion via PI3K/AKT/mTOR signaling pathway-mediated glycolysis

**Fig. 8:**
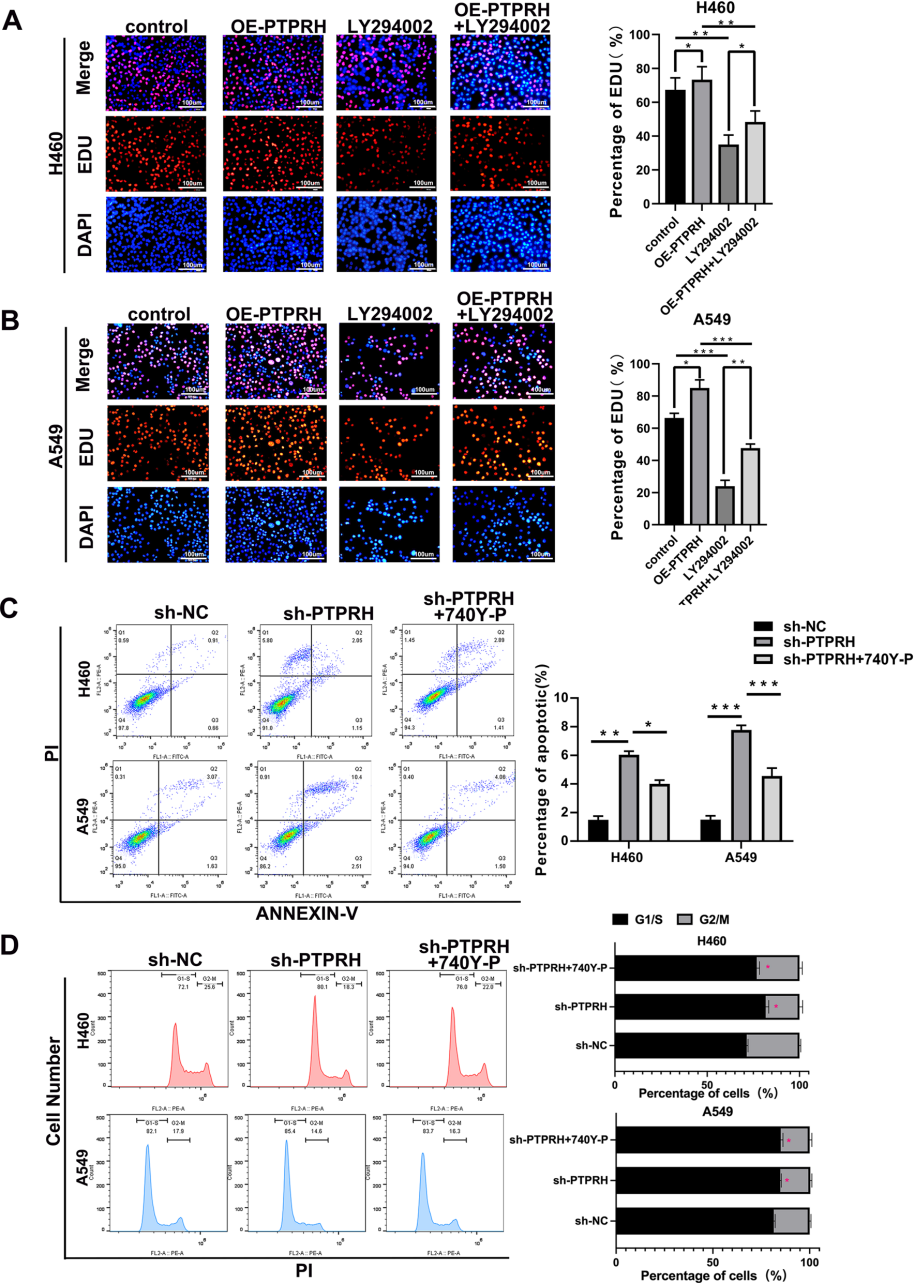
PTPRH promoted proliferation and invasion via PI3K/AKT/mTOR signaling pathway-mediated glycolysis. **A**, **B** EdU assay to verify the individual and combined effects of sh-PTPRH and the PI3K inhibitor LY294002 on the number of cells undergoing DNA replication. **C**, **D** Flow cytometry assays to detect the reversal of the proapoptotic effect of sh-PTPRH after treatment with the PI3K activator 740Y-P in H460 and A549 cells

**Fig. 9:**
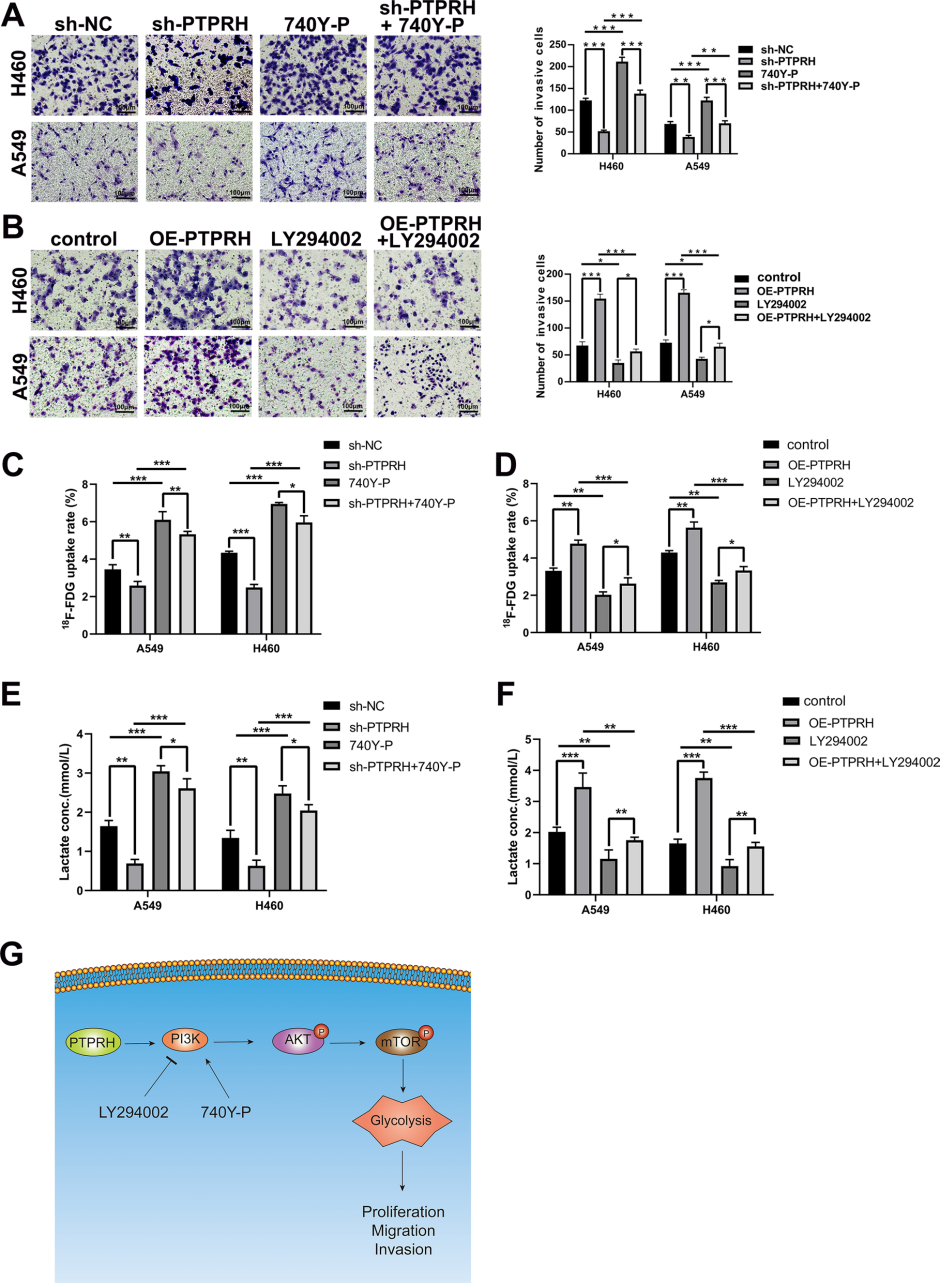
PTPRH increased invasion, FDG uptake and lactate levels via PI3K/AKT/mTOR signaling pathway-mediated glycolysis. **A**, **B** Transwell assays to detect the individual and combined effects of sh-PTPRH and the PI3K activator 740Y-P and OE-PTPRH and the PI3K inhibitor LY294002 on the invasion ability of H460 and A549 cells. **C**, **D** ^18^F-FDG uptake assays to detect the individual and combined effects of sh-PTPRH and the PI3K activator 740Y-P and OE-PTPRH and the PI3K inhibitor LY294002 on the FDG uptake ability of H460 and A549 cells. **E**, **F** Cell metabolism assays to detect the individual and combined effects of sh-PTPRH and the PI3K activator 740Y-P and OE-PTPRH and the PI3K inhibitor LY294002 on the lactate levels of H460 and A549 cells. **G** A diagram showing the possible mechanism underlying the modulation of glycolysis by PTPRH

Our previous study showed that PTPRH regulates glycolysis through the PI3K/AKT/mTOR signaling pathway. We simultaneously interfered with PTPRH expression and used an inhibitor and activator of PI3K to verify cell proliferation and invasion in vitro. First, the results of EdU experiments showed that the number of cells in the DNA replication stage was relatively increased after overexpression of PTPRH in both A549 and H460 cells and was significantly downregulated after treatment with LY294002. The effect after overexpression of PTPRH was reversed after LY294002 addition (Fig. 8A, B). In addition, flow cytometry analysis showed that apoptosis increased in both H460 and A549 cells after PTPRH was knocked down alone. However, increased apoptosis caused by sh-PTPRH was reversed when sh-PTPRH and 740Y-P were added simultaneously (Fig. 8C, D). The results of the Transwell assay showed that in H460 and A549 cells, the number of invasive cells was reduced after PTPRH was knocked down, the number of invasive cells was increased after the use of 740Y-P, and the decrease in invasive cells caused by sh-PTPRH was reversed when sh-PTPRH and 740Y-P were added simultaneously (Fig. 9A). Similarly, a rescue experiment was performed to verify this trend in the OE-PTPRH and LY294002 groups (Fig. 9B). ^18^F-FDG uptake assay detected that the FDG uptake rate was lower in sh-PTPRH group, 740Y-P group had increased FDG uptake rate, and the decreased FDG uptake rate after PTPRH was knocked down was reversed when sh-PTPRH and 740Y-P were added simultaneously (Fig. 9C). Similarly, a rescue experiment was performed to verify this trend in the OE-PTPRH and LY294002 groups (Fig. 9D). After PTPRH was knocked down, lactate level had decreased, lactate levels were increased after the use of 740Y-P, and lower lactate levels caused by sh-PTPRH were reversed when sh-PTPRH and 740Y-P were added simultaneously (Fig. 9E). Similarly, a rescue experiment was performed to verify this trend in the OE-PTPRH and LY294002 groups (Fig. 9F). These results suggested that PTPRH promotes glycolysis, proliferation, migration, and invasion via the PI3K/AKT/mTOR signaling pathway in NSCLC. A possible mechanism underlying the modulation of glycolysis by PTPRH is shown in Fig. 9G.

## Discussion

The incidence rates of malignant tumors and lung cancers and the associated mortality rates are increasing [1]. Although great progress has been made in treating lung cancer, the overall prognosis is still not ideal because of its complex clinical manifestations and substantial heterogeneity [2]. The most common pathological type of lung cancer is NSCLC [29]. Therefore, there is a crucial need to conduct in-depth research on the mechanisms underlying NSCLC progression and to explore new key targets. Targeted tumor energy metabolism therapy has received considerable attention and has potential clinical significance.

Tumor cells obtain energy through glycolysis in an aerobic environment, a process called aerobic glycolysis. Targeting aerobic glycolysis is a new approach to tumor treatment. Mutations in the *PTPRH* gene are the most common mutations in the protein tyrosine phosphatase family [23]. Previous studies have shown that PTPRH can reduce protein phosphorylation through related signaling pathways to alleviate airway obstruction in patients with asthma [30]. In recent years, studies have shown that PTPRH regulates the occurrence and development of cancer [24–26], but the correlation of PTPRH expression with glycolysis is still unclear. This study focused on the biological role and mechanism of PTPRH in promoting NSCLC energy metabolism, migration, and invasion via the PI3K/AKT/mTOR signaling pathway.

The expression of PTPRH in NSCLC is significantly higher than that in normal tissues based on an analysis of datasets from TCGA and GEO (Fig. 1). This is consistent with the findings of Chen et al. and Sato et al. [25, 26]. We performed immunohistochemical staining of PTPRH in 80 primary lesion tissue samples from NSCLC patients and 40 matched adjacent lung tissue samples. The results showed that PTPRH expression was significantly higher in the primary lesions of patients with NSCLC (Fig. 2A, B). Furthermore, a study was conducted on the correlation between PTPRH expression in the primary lesion tissue samples of 80 patients with NSCLC and clinical data. PTPRH expression was closely correlated with the tumor diameter and clinical stage of NSCLC patients but not with the age and sex of patients with NSCLC (Table 1). This result suggests that the differential expression of PTPRH in NSCLC has clinical significance, but its correlation with glycolysis in NSCLC remains unclear. Given the increased research focus on tumor metabolic abnormalities, the impact of PTPRH on the biological behavior and glycolysis of tumor cells has potential clinical applications.

Aerobic glycolysis is an important feature of energy metabolism in tumor cells [20] and refers to the ability of malignant tumors to obtain adenosine triphosphate through glycolysis under aerobic conditions, also known as the Warburg effect [31, 32]. This effect is the molecular basis for the use of ^18^F-FDG PET/CT in oncology [4, 5, 33]. The glucose metabolism level of tumor cells can be reflected by ^18^F-FDG PET/CT imaging of indicators of glucose metabolism. Immunohistochemical staining of PTPRH was performed on tissue slices from 80 patients with NSCLC. Correlation analysis showed that PTPRH expression in cancer tissues was correlated with SUVmax, MTV, and TLG (Fig. 2E). We also conducted a correlation analysis between the expression levels of PTPRH and glycolysis-related proteins (GLUT1, HK2, PKM2, and LDHA), and the results revealed a correlation (Fig. 2E). Therefore, we speculated that PTPRH could be involved in glycolysis in NSCLC, but its specific molecular mechanism required further exploration.

The specific molecular mechanism underlying PTPRH participation in glycolysis in NSCLC was explored further based on the results of the first part of the study. First, by performing RT‒PCR and western blotting of the A549, H1299, H292, PC9, Calu-1, H460, and HCC827 NSCLC cell lines and the HBE human bronchial epithelial cell line, we found that PTPRH was highly expressed to varying degrees in NSCLC cell lines (Fig. 3A, B). Although the expression of PTPRH varies in different tumors [34], the differential expression of PTPRH observed in this study is consistent with the analysis of histological specimens and with the research results of Chen et al. and Sato et al. [25, 26]. In vitro, we verified through colony formation, MTT, and EdU assays that PTPRH increases cell proliferation (Fig. 3E–H). Transwell and scratch assays confirmed that PTPRH improves cell invasion and migration abilities (Fig. 4), and flow cytometry confirmed that downregulation of PTPRH can promote cell apoptosis (Fig. 5C, D). However, a G1/S phase cell cycle blockade was observed after PTPRH downregulation (Fig. 5E, F). In vivo, we used micro-PET scans with ^18^F-FDG and found that after downregulation of PTPRH, the ^18^F-FDG SUVmax values of the tumors significantly decreased (Fig. 6E). This trend was validated by the immunohistochemistry results (Fig. 6G). ^18^F-FDG uptake and lactate experiments were performed to further explore the relationship between PTPRH and glycolysis (Fig. 6H–I). Western blotting results showed that PTPRH increased the expression of GLUT1, HK2, PKM2, and LDHA, confirming the hypothesis that PTPRH promotes glycolysis in NSCLC (Fig. 6J).

Gene set enrichment analysis (GSEA) revealed that the differentially expressed gene *PTPRH* was enriched in KEGG pathways such as E2F, glycolysis, PI3K/AKT/mTOR, and apoptosis (Fig. 7A). Based on the results of GSEA, while PI3K/AKT/mTOR signaling pathway also has multiple functions in the regulation of various biological behaviors in tumor cells and plays a crucial role in the occurrence and development of NSCLC [35]. This signaling pathway was thus selected for further validation and mechanistic studies. Western blotting confirmed that the downregulation and upregulation of PTPRH can inhibit and promote the expression of related proteins in the PI3K/AKT/mTOR signaling pathway, which was validated using the PI3K agonist 740Y-P and inhibitor LY294002 (Fig. 7C, D). The response experiment confirmed that PTPRH affected glycolysis-related protein expression (Fig. 7E, F) and cell behavior (Figs. 8, 9) via the PI3K/AKT/mTOR signaling pathway. In conclusion, PTPRH affects glycolysis in NSCLC cells via the PI3K/AKT/mTOR signaling pathway and ultimately promotes tumor progression in NSCLC, which could be regulated by LY294002 and 740Y-P. However, further exploration is needed to determine whether PTPRH can regulate potential intermediate targets and reduce the expression of inhibitory factors of the PI3K/AKT/mTOR signaling pathway, thereby enhancing the effect of the pathway. Further exploration of the mechanism by which PTPRH expression regulates glucose metabolism is expected to provide a new therapy targeting energy metabolism in NSCLC. We also plan to further explore the use of PTPRH-targeting compounds or gene therapy in NSCLC xenograft models.

## Conclusions

In summary, we report that PTPRH promotes glycolysis, proliferation, migration, and invasion via the PI3K/AKT/mTOR signaling pathway in NSCLC and ultimately promotes tumor progression, which can be regulated by LY294002 and 740Y-P. These results suggest that PTPRH is a potential therapeutic target for NSCLC.

### Supplementary Information

**Additional file 1.** GSEA of the single-gene KEGG pathway for PTPRH and Western blotting statistical images of rescue experiment.

## Data Availability

All experimental datasets generated for this study are included in the article/ Additional file.

## Abbreviations

^18^F-FDG: ^18^F-fluorodeoxyglucose
CT: Computed tomography
GEO: Gene Expression Omnibus
GLUT1: Glucose transporter type 1
GSEA: Gene Set Enrichment Analysis
HK2: Hexokinase 2
KEGG: Kyoto Encyclopedia of Genes and Genomes
LDHA: Lactate dehydrogenase A
MTV: Metabolic tumor volume
NSCLC: Non-small cell lung cancer
PET: Positron emission tomography
PI3K: Phosphatidylinositol-3-kinase
PKM2: Pyruvate kinase M2
PTP: Protein tyrosine phosphatase
PTPRH: Protein tyrosine phosphatase H receptor
RTK: Receptor tyrosine kinase
SUVmax: Maximum standard uptake value
SUVmean: Mean standard uptake value
TCGA: The Cancer Genome Atlas
TLG: Total lesion glycolysis

## References

[1] Siegel RL, Miller KD, Fuchs HE, Jemal A (2022). Cancer statistics, 2022. Cancer J Clin.

[2] Wood DE, Kazerooni EA, Baum SL, Eapen GA, Ettinger DS, Hou L, Jackman DM, Klippenstein D, Kumar R, Lackner RP (2018). Lung cancer screening, Version 3.2018, NCCN clinical practice guidelines in oncology. J Natl Compr Cancer Netw..

[3] Kim S, Im JH, Kim WK, Choi YJ, Lee JY, Kim SK, Kim SJ, Kwon SW, Kang KW (2021). Enhanced sensitivity of nonsmall cell lung cancer with acquired resistance to epidermal growth factor receptor-tyrosine kinase inhibitors to phenformin: the roles of a metabolic shift to oxidative phosphorylation and redox balance. Oxid Med Cell Longev.

[4] Warburg O (1956). On respiratory impairment in cancer cells. Science.

[5] Liao X, Liu M, Wang R, Zhang J (2021). Potentials of non-invasive (18)F-FDG PET/CT in immunotherapy prediction for non-small cell lung cancer. Front Genet.

[6] Ntziachristos V, Pleitez MA, Aime S, Brindle KM (2019). Emerging technologies to image tissue metabolism. Cell Metab.

[7] Salas JR, Clark PM (2022). Signaling pathways that drive (18)F-FDG accumulation in cancer. J Nucl Med.

[8] Xu S, Herschman HR (2019). A tumor agnostic therapeutic strategy for hexokinase 1-Null/Hexokinase 2-positive cancers. Can Res.

[9] Zhu S, Guo Y, Zhang X, Liu H, Yin M, Chen X, Peng C (2021). Pyruvate kinase M2 (PKM2) in cancer and cancer therapeutics. Cancer Lett.

[10] Sharma D, Singh M, Rani R (2022). Role of LDH in tumor glycolysis: regulation of LDHA by small molecules for cancer therapeutics. Semin Cancer Biol.

[11] Robey RB, Hay N (2009). Is Akt the "Warburg kinase"?-Akt-energy metabolism interactions and oncogenesis. Semin Cancer Biol.

[12] Harsha C, Banik K, Ang HL, Girisa S, Vikkurthi R, Parama D, Rana V, Shabnam B, Khatoon E, Kumar AP (2020). Targeting AKT/mTOR in oral cancer: mechanisms and advances in clinical trials. Int J Mol Sci.

[13] Hoxhaj G, Manning BD (2020). The PI3K-AKT network at the interface of oncogenic signalling and cancer metabolism. Nat Rev Cancer.

[14] Saraon P, Pathmanathan S, Snider J, Lyakisheva A, Wong V, Stagljar I (2021). Receptor tyrosine kinases and cancer: oncogenic mechanisms and therapeutic approaches. Oncogene.

[15] Sudhesh Dev S, Zainal Abidin SA, Farghadani R, Othman I, Naidu R (2021). Receptor tyrosine kinases and their signaling pathways as therapeutic targets of curcumin in cancer. Front Pharmacol.

[16] Jiang Y, Zeng Q, Jiang Q, Peng X, Gao J, Wan H, Wang L, Gao Y, Zhou X, Lin D (2022). (18)F-FDG PET as an imaging biomarker for the response to FGFR-targeted therapy of cancer cells via FGFR-initiated mTOR/HK2 axis. Theranostics.

[17] Jin N, Bi A, Lan X, Xu J, Wang X, Liu Y, Wang T, Tang S, Zeng H, Chen Z (2019). Identification of metabolic vulnerabilities of receptor tyrosine kinases-driven cancer. Nat Commun.

[18] Li J, Lu H, Ng PK, Pantazi A, Ip CKM, Jeong KJ, Amador B, Tran R, Tsang YH, Yang L (2022). A functional genomic approach to actionable gene fusions for precision oncology. Sci Adv.

[19] Mrozek EM, Bajaj V, Guo Y, Malinowska IA, Zhang J, Kwiatkowski DJ (2021). Evaluation of Hsp90 and mTOR inhibitors as potential drugs for the treatment of TSC1/TSC2 deficient cancer. PLoS ONE.

[20] Vaupel P, Schmidberger H, Mayer A (2019). The Warburg effect: essential part of metabolic reprogramming and central contributor to cancer progression. Int J Radiat Biol.

[21] Sarmento-Ribeiro AB, Scorilas A, Gonçalves AC, Efferth T, Trougakos IP (2019). The emergence of drug resistance to targeted cancer therapies: clinical evidence. Drug Resist Updates.

[22] Sivaganesh V, Sivaganesh V, Scanlon C, Iskander A, Maher S, Lê T, Peethambaran B (2021). Protein tyrosine phosphatases: mechanisms in cancer. Int J Mol Sci.

[23] Frankson R, Yu ZH, Bai Y, Li Q, Zhang RY, Zhang ZY (2017). Therapeutic targeting of oncogenic tyrosine phosphatases. Can Res.

[24] Bujko M, Kober P, Statkiewicz M, Mikula M, Grecka E, Rusetska N, Ligaj M, Ostrowski J, Siedlecki JA (2017). Downregulation of PTPRH (Sap-1) in colorectal tumors. Int J Oncol.

[25] Chen A, Ding S, Shen X, Lin X (2021). The high expression of PTPRH is associated with poor prognosis of human lung adenocarcinoma. Comput Math Methods Med.

[26] Sato T, Soejima K, Arai E, Hamamoto J, Yasuda H, Arai D, Ishioka K, Ohgino K, Naoki K, Kohno T (2015). Prognostic implication of PTPRH hypomethylation in non-small cell lung cancer. Oncol Rep.

[27] Nunes-Xavier CE, Aurtenetxe O, Zaldumbide L, López-Almaraz R, Erramuzpe A, Cortés JM, López JI, Pulido R (2019). Protein tyrosine phosphatase PTPN1 modulates cell growth and associates with poor outcome in human neuroblastoma. Diagn Pathol.

[28] Wang S, Zhang H, Du B, Li X, Li Y (2022). Fuzzy planar cell polarity gene (FUZ) promtes cell glycolysis, migration, and invasion in non-small cell lung cancer via the phosphoinositide 3-kinase/protein kinase B pathway. J Cancer.

[29] Poomakkoth N, Issa A, Abdulrahman N, Abdelaziz SG, Mraiche F (2016). p90 ribosomal S6 kinase: a potential therapeutic target in lung cancer. J Transl Med.

[30] Chen FJ, Du LJ, Zeng Z, Huang XY, Xu CY, Tan WP, Xie CM, Liang YX, Guo YB (2022). PTPRH alleviates airway obstruction and Th2 inflammation in asthma as a protective factor. J Asthma Allergy.

[31] Fukushi A, Kim HD, Chang YC, Kim CH (2022). Revisited metabolic control and reprogramming cancers by means of the warburg effect in tumor cells. Int J Mol Sci.

[32] Jacquet P, Stéphanou A (2022). Searching for the metabolic signature of cancer: a review from Warburg's time to now. Biomolecules.

[33] Vanhove K, Thomeer M, Derveaux E, Shkedy Z, Owokotomo OE, Adriaensens P, Mesotten L (2019). Correlations between the metabolic profile and (18)F-FDG-Positron Emission Tomography-Computed Tomography parameters reveal the complexity of the metabolic reprogramming within lung cancer patients. Sci Rep.

[34] Kim M, Ryu SE (2022). Crystal structure of the catalytic domain of human RPTPH. Acta Crystallographica Section F, Struct Biol Commun.

[35] Iksen, Pothongsrisit S, Pongrakhananon V (2021). Targeting the PI3K/AKT/mTOR signaling pathway in lung cancer: an update regarding potential drugs and natural products. Molecules.

